# Subsurface geology detection from application of the gravity-related dimensionality constraint

**DOI:** 10.1038/s41598-024-52843-5

**Published:** 2024-01-30

**Authors:** Kurosh Karimi, Gunther Kletetschka

**Affiliations:** 1 Institute of Hydrogeology, Engineering Geology and Applied Geophysics, Faculty of Science, Charles University, Prague, 12843 Czech Republic; 2 Geophysical Institute, University of Alaska-Fairbanks, 903 N Koyukuk Drive, Fairbanks, AK 99709 USA

**Keywords:** Geology, Geophysics

## Abstract

Geophysics aims to locate bodies with varying density. We discovered an innovative approach for estimation of the location, in particular depth of a causative body, based on its relative horizontal dimensions, using a dimensionality indicator (*I*). The method divides the causative bodies into two types based on their horizontal spread: line of poles and point pole (LOP–PP) category, and line of poles and plane of poles (LOP–POP) category; such division allows for two distinct solutions. The method’s depth estimate relates to the relative variations of the causative body’s horizontal extent and leads to the solutions of the Euler Deconvolution method in specific cases. For causative bodies with limited and small depth extent, the estimated depth (z^^^_0_) corresponds to the center of mass, while for those with a large depth extent, z^^^_0_ relates to the center of top surface. Both the depth extent and the dimensionality of the causative body influence the depth estimates. As the depth extent increases, the influence of *I* on the estimated depth is more pronounced. Furthermore, the behavior of z^^^_0_ exhibits lower errors for larger values of *I* in LOP–POP solutions compared with LOP–PP solutions. We tested several specific model scenarios, including isolated and interfering sources with and without artificial noise. We also tested our approach on real lunar data containing two substantial linear structures and their surrounding impact basins and compared our results with the Euler deconvolution method. The lunar results align well with geology, supporting the effectiveness of this approach. The only assumption in this method is that we should choose between whether the gravity signal originates from a body within the LOP–PP category or the LOP–POP category. The depth estimation requires just one data point. Moreover, the method excels in accurately estimating the depth of anomalous causative bodies across a broad spectrum of dimensionality, from 2 to 3D. Furthermore, this approach is mathematically straightforward and reliable. As a result, it provides an efficient means of depth estimation for anomalous bodies, delivering insights into subsurface structures applicable in both planetary and engineering domains.

## Introduction

A primary objective of potential field survey is to locate geological bodies beneath the surface^1^. Depth estimation of causative bodies with contrasting magnetization and/or density ranges from a simple graphical approaches^2–4^ to more sophisticated inverse problems^5–14^. In an influential paper, Thompson^7^ introduced a location estimation technique based on Euler’s homogeneity relationship, enabling the investigation of subsurface magnetic anomalies with varying shapes. The location estimation of the gravity anomalies using Euler’s homogeneity equation was later considered by others (e.g., Reid et al.^9^). The advent of Marussi tensor (e.g., Pedersen and Rasmussen)^15^, also known as the gravity gradient tensor (GGT), improved the depth and edge detection of gravitationally anomalous structures to a great extent. GGT extracts information from geologic bodies with contrasting density relative to their surroundings. This tensor, in itself, is a powerful tool to give quantitative information about the strike direction^13,16,17^, horizontal dimensions^18^, structural weakness^16,17^, and locations^1,7^ related to subsurface anomalous constructs. It applies in both engineering and planetary exploration, and has been extensively used for detecting local petroleum, metal, diamond, and groundwater^1,13,15,19–21^, as well as for exploration of the Earth’s crus^18,20,22,23^, and celestial bodies^16,24–26^. Nowadays, the gravity data obtained from satellites have significantly improved in precision and ground resolution, enabling the GGT to explore planetary properties in more detail than ever before^27^.

Pedersen and Rasmussen^15^ conducted an in-depth study of GGT, including its invariants, eigenvalues, and eigenvectors, which was later expanded and used by Marson and Klingele^28^, Zheng et al*.*^11^, Mikhailov et al*.*^12^, Beiki and Pedersen^13^ and Zuo et al*.*^29^ for depth estimation of gravity anomalies. Zhang et al.^11^ and Mikhailov et al*.*^12^ showed that, instead of the gravity anomaly, the components of the GGT can be used to improve the Euler deconvolution method. Beiki and Pedersen^13^ developed a new technique to depth estimate of geological structures using the eigenvector analysis of the GGT. Wedge^30^ created an algorithm, using an accumulation method to identify potential mass anomalies by casting lines through a volume based on GGT properties and accumulating votes. Local maxima in the volume, in his algorithm, correspond to mass anomalies. Zhou^31^ extended the normalized imaging method to interpret GGT data and normalized directional analytic signals and calculated the salt dome structure’s horizontal position and depth range. Zuo et al.^29^ introduced a method using eigenvector analysis for locating the centroids and horizontal boundaries of geological structures from GGT. The method employed eigenvector analysis to extract source centroid information, differing from traditional potential field boundary detection. This discussed theoretical foundations and the physical significance of GGT eigenvector analysis and addressed challenges related to multiple sources and parameter identification. Yuan et al*.*^32^ came with a novel depth estimation method based on the Chebyshev–Padé downward continuation technique, emphasizing stability and depth calculation; the research compared filter curves in the wave number domain between Tikhonov regularization and Chebyshev–Padé methods, highlighting the convergence of the latter’s filter curve to zero, making it suitable for depth estimation. Zhou et al*.*^14^ improved the normalization imaging method using the downward continuation method based on continued fractions, obtaining the source geometric parameters under the source distribution at different depths.

In parallel, the magnetic gradient tensor^33,34^, extracted from the directional derivatives of the magnetic field anomaly has also been utilized by some workers. However, the magnetic gradient tensor is less informative than its gravity analogue, due to the complex nature of the magnetic anomaly field.

The dimensionality indicator (*I*), varying from 0 to 1, is a quantitative index used to indicate the relative magnitudes of the horizontal dimensions of a causative body^15^. It is determined when formulating the characteristic equation to calculate the eigenvalues of the GGT. From a geophysical standpoint, a causative body can be categorized as pure two–dimensional (2D), pure three–dimensional (3D), or somewhere in between (2–3D). The dimensionality indicator for a pure 2D, pure 3D and 2–3D causative body is defined as follows^15^:

- Pure 2D (*I* = *0*): One horizontal dimension goes to physical infinity and becomes much larger than the other horizontal dimension in practice.
- Pure 3D (*I* = *1*): The two horizontal dimensions of the body are identical.
- 2–3D ($$0<I<1)$$: One horizontal dimension is larger than the other, but the dimensions are still comparable.

The dimensionality classification strongly depends on the dimensions of the causative body, the measurement point distance from the body, and the resolution of the grid unit^26^. While the usage of *I* has been limited to constraining the relative horizontal dimensions of subsurface anomalous structures^12,13,20^ to show whether it is 3D or 2D, we show in this research that further information could be extracted from this parameter. Though Mikhailov et al*.*^12^ used *I* to calculate the depth of two simple-shaped gravity anomalies: the point pole (*I* = *1*) and the line of poles (*I* = *0*), they did not provide any solutions for cases where *I* is neither zero nor one. They considered the structural index (SI) in Euler deconvolution method as 1 + *I*, whereby set up SI = 1 for a line of poles and SI = 2 for a point pole. Here, we fill this research gap and outline a new method for detecting the depth of geological constructs with a spectrum of dimensionality indicator from zero to one. We test the effect of depth extent on the solutions and examine synthetic data with isolated and interfering different shape sources in the presence and absence of 5% random Gaussian noise. While this method can be applied in engineering domains, ranging from micro-gravimetry to ordinary ground and airborne gravimetry, we choose to demonstrate its application on real lunar data at a planetary scale. For this purpose, we use the Bouguer potential, obtained by subtracting the topographic gravity potential from the measured gravity potential, represented by spherical harmonic series (SHS). The applied gravity model for the Moon is “GRGM1200A”^35^, truncated at d/o = 600 and continued downward to an elevation of 5 km with respect to the reference ellipsoid. The truncation and continuation serve to minimize the artifacts due to frequency aliasing^35^. The grid resolution of GRGM1200A at d/o = 600 is approximately 10 km on the ground with a precision of around 10 mGal^19^. The utilized topographic gravity model “STU_MoonTopo720”^36^, was truncated at a degree and order of 600 to be consistent with the measure gravity model. The Bouguer-type $\Gamma$ components are extracted using Graflab software^37^. Our methodology, when applied to real data, is compared with the Euler deconvolution method, and our solutions fall within the range of solutions provided by Euler deconvolution.

An important point regarding the components of GGT is that (1) they can be directly calculated by taking the second directional derivatives of the gravity potential in the spatial domain, (2) they can be obtained by taking derivatives in the Fourier domain and then transforming back to the spatial domain, and (3) they can be measured directly by gradiometers, on board the ships, airplanes and on the ground. In the case of gradiometry, the error effect is even less significant because of directly measured (not computed) components of GGT^13^.

## Theory

We present a formula utilizing the components of GGT to estimate the distance to an unknown causative body underground (z_0_). When the causative body has a limited depth extent, the estimated depth (z^^^_0_) corresponds to the depth of the center of mass (COM); conversely, for vertically extended bodies, z^^^_0_ represents an estimation of the center of top surface (COTS).

Due to the non-uniqueness of the gravity data, determining the precise geometry of a causative body that generates the gravity anomaly field is impossible^1^. In other words, numerous models can produce the same gravity response. Nonetheless, the lack of uniqueness can be addressed by considering hypotheses about the shape of the density transition. In fact, it is theoretically more advantageous to work with a generalized geophysical hypothesis and arrive at a single solution, rather than accepting a solution that could be significantly divergent from reality due to adherence to a purely mathematical criterion (as discussed in Sanso et al.)^38^. Considering a priori known geometry, the horizontal extent of the body can also be inferred from the gravity data.

The GGT, ${\varvec{\Gamma}}$, is defined as^15^.

1 $${\varvec{\Gamma}}= \nabla (\nabla \mathrm{\rm T})=\left[\begin{array}{ccc}{\Gamma }_{ii}& {\Gamma }_{ij}& {\Gamma }_{ik}\\ {\Gamma }_{ji}& {\Gamma }_{jj}& {\Gamma }_{jk}\\ {\Gamma }_{ki}& {\Gamma }_{kj}& {\Gamma }_{kk}\end{array}\right]$$

where T is disturbing gravity potential, subscripts *ij* are two orthogonal components of the coordinate system (e.g., in Cartesian coordinates each of “*i*”, “*j*” and “*k*” are x, y, z). The *ith* component of the gravity vector is ${g}_{i}=\frac{\partial T}{\partial i}$ and the second derivative components of the gravity potential are ${\Gamma }_{ij}=\frac{\partial }{\partial i}\left({g}_{j}\right)= \frac{\partial }{\partial i}\left(\frac{\partial T}{\partial j}\right)= \frac{{\partial }^{2}T}{\partial i \partial j}={\Gamma }_{ji}$.

There are only five independent components in Eq. (1) for two reasons: (1) In a free source condition (that is when the measurement is conducted out of the gravitational source, from satellite, airplane or on the Earth surface), the Laplace equation holds, i.e., ${\nabla }^{2} \mathrm{\rm T}=0\,and\,{\Gamma }_{kk}=-({\Gamma }_{ii}+{\Gamma }_{jj})$, and (2) In as much as $\nabla \times \mathop{g}\limits^{\rightharpoonup} = 0, {{\varvec{\Gamma}}}$ is symmetric (${\Gamma }_{ik}={\Gamma }_{ki}, {\Gamma }_{ij}={\Gamma }_{ji}, \,and\, {\Gamma }_{jk}={\Gamma }_{kj}$).

${\varvec{\Gamma}}$ has three invariants *I*_*0*_*, I*_*1*_ and *I*_*2*_, meaning that under any coordinate rotation, their values do not change^15^:

$${I}_{0}=Trace\left({\varvec{\Gamma}}\right)=\sum_{i=1}^{3}{\Gamma }_{ii}=0$$

2 $${I}_{1}=\frac{1}{2}\left((Trace {\left({\varvec{\Gamma}}\right))}^{2}-Trace({{\varvec{\Gamma}}}^{2})\right)={\Gamma }_{ii}{\Gamma }_{jj}+{\Gamma }_{ii}{\Gamma }_{kk}+{\Gamma }_{jj}{\Gamma }_{kk}-{{\Gamma }_{ij}}^{2}-{{\Gamma }_{jk}}^{2}-{{\Gamma }_{ik}}^{2}$$

3 $${I}_{2}=det\left({\varvec{\Gamma}}\right)={\Gamma }_{ii}\left({\Gamma }_{jj}{\Gamma }_{kk}- {\Gamma }_{jk}{\Gamma }_{kj}\right)+{\Gamma }_{ij}\left({\Gamma }_{jk}{\Gamma }_{ki} - {\Gamma }_{ji}{\Gamma }_{kk}\right)+{\Gamma }_{ik}\left({\Gamma }_{ji}{\Gamma }_{kj}- {\Gamma }_{jj}{\Gamma }_{ki}\right)$$

Since ${\varvec{\Gamma}}$ is a symmetric matrix, its eigenvectors and eigenvalues should be perpendicular and real, respectively. Thus, it follows that the dimensionality indicator (*I*) be^15^:

4 $$0\le I=-\frac{{{(I}_{2}/2)}^{2}}{{{(I}_{1}/2)}^{3}}\le 1$$

*Γ*_*zz*_, *I*_*1*_, and *I*_*2*_ are three parameters that amplify the anomalous sources that are near the surface and/or small. In other words, the signals from geological structures, whether deep or broad, are attenuated by these parameters. The unit of *Γ*_*zz*_ is acceleration divided by distance ((m/s^2^)/m = s^−2^). According to relations (2) and (3), the units of *I*_*1*_ and *I*_*2*_ are s^–4^ and s^–6^, respectively.

In potential field data, the signal from the causative body falls off as a function of the inverse of its distance from the observation point^6^. That is, the signal from the upper parts is stronger than the lower parts of the body in *g*_*z*_ parameter. Additionally, the signals arising from the shallow parts are amplified by *Γ*_*zz*_ (This is because *Γ*_*zz*_ is obtained by the multiplication of wavenumber in *g*_*z*_ in the Fourier domain, gaining higher weights by increasing wave number or getting closer to the surface). Since in estimating the depth of anomalous structures, we employ *g*_*z*_ and *Γ*_*zz*_, it stands to reason that the upper parts have a major contribution to the received signals. Therefore, depending on the depth extent of the body, the z^^^_0_ could represent the COM in the case of limited and small depth extent, or the COTS if the depth extent is extended.

**Figure 1:**
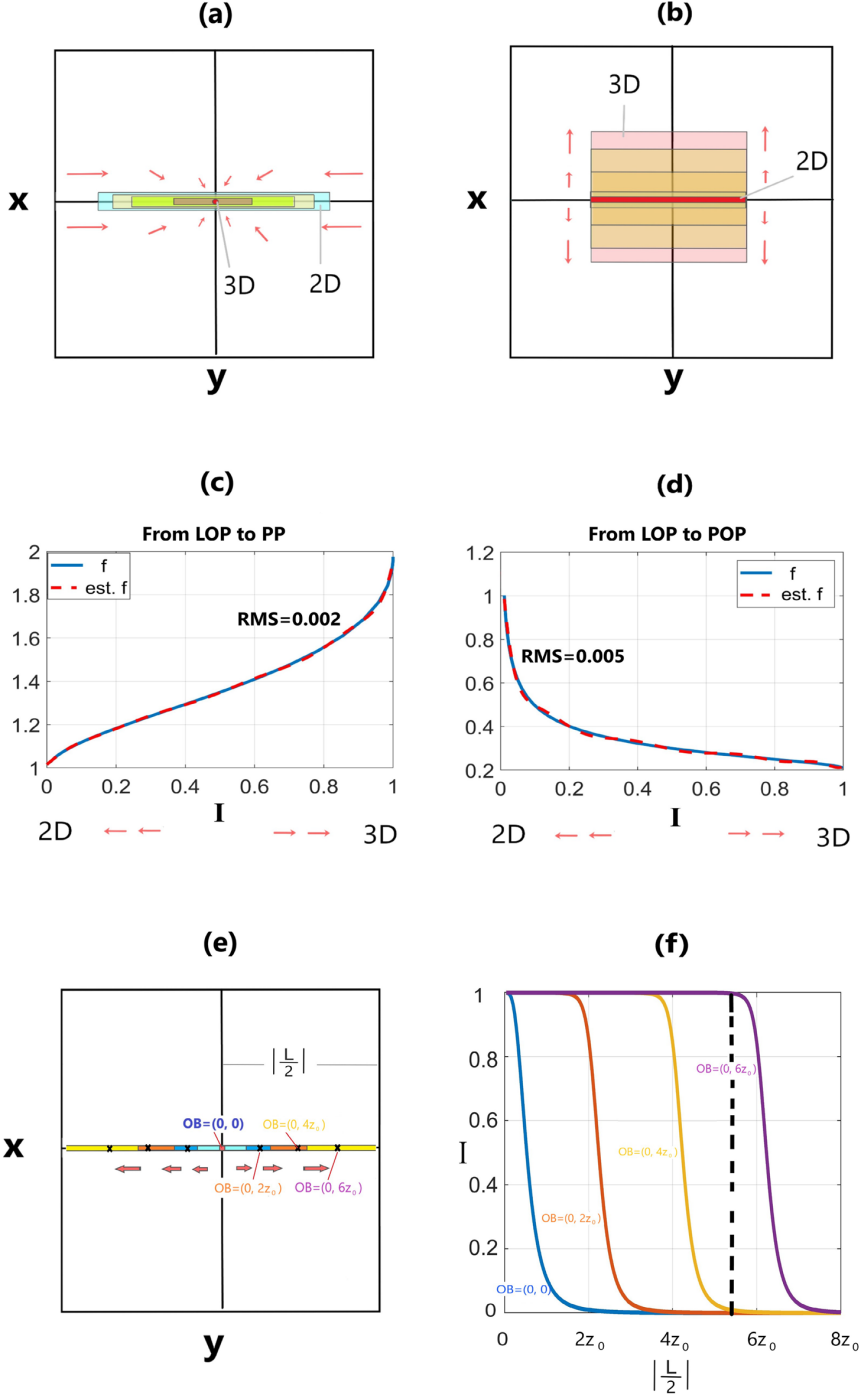
variations of a causative body (**a**) from line of poles (LOP) (pure 2D) to point pole (PP) (pure 3D); (**b**) from line of poles to plane of poles (POP) (pure 3D); variations of $f$ with respect to *I* when the body changes from (**c**) LOP to PP; (**d**) from LOP to POP. The red dashed lines in “c” and “d” are 10th order polynomial, estimating $f$ in terms of *I*. (**e**) variations from LOP to PP with observation points (OB) with (x, y) coordinates at (0, 0), (0, $2{z}_{0}$), (0, $4{z}_{0}$), and (0, $6{z}_{0}$) where z_0_ is the depth of the causative body (y is in terms of depth); (**f**) variations of *I* at different observation points when the length changes from zero to physical infinity ($|\frac{L}{2}|=8{z}_{0}$). The dashed black line in Figure f demonstrates the condition in which the length of the LOP is fixed but the observation point changes.

Here, we consider prismatic bodies as models, which can represent various geological features. We explore two types of models, those that lie between a line of poles and a point pole (LOP–PP) (Fig. 1a), and those that lie between a line of poles and a plane of poles (LOP–POP) (Fig. 1b). Depending on the horizontal extent of the anomalous mass, the user can choose between the two categories of solutions explained further in the next sections.

### Simulated data

#### 2–3D models

When dealing with a causative body that does not maintain a balance between its horizontal dimensions, implying it is 2–3D in nature, it can be effectively modelled using a rectangular prism. This prism is characterized by dimensions $\Delta x={x}_{2}-{x}_{1},\Delta y={y}_{2}-{y}_{1}, {\text{and}}\Delta z={z}_{2}-{z}_{1}$ . The disturbing gravity vector, $$\mathbf{g}=\left[\begin{array}{c}{{\text{g}}}_{x}\\ {{\text{g}}}_{y}\\ {{\text{g}}}_{z}\end{array}\right]$$, of this prismatic construct in a Cartesian coordinates system is^39^:

$${\left({{\text{g}}}_{x}\right)}_{2-3D}=GM{\sum }_{i=1}^{2}{\sum }_{j=0}^{2}{\sum }_{k=0}^{2}{\mu }_{ijk}\left[ X{\text{arctan}}\left(\frac{Z Y}{X {R}_{ijk}}\right)- Z{\text{ln}}\left({R}_{ijk}+Y\right)- Y{\text{ln}}\left({R}_{ijk}+Z\right)\right]$$

$${\left({{\text{g}}}_{{\text{y}}}\right)}_{2-3D}=GM{\sum }_{i=1}^{2}{\sum }_{j=0}^{2}{\sum }_{k=0}^{2}{\mu }_{ijk}\left[ Y{\text{arctan}}\left(\frac{X Z}{Y {R}_{ijk}}\right)- X{\text{ln}}\left({R}_{ijk}+Z\right)- Z {\text{ln}}({R}_{ijk}+X)\right]$$

5 $${\left({{\text{g}}}_{z}\right)}_{2-3D}=GM{\sum }_{i=1}^{2}{\sum }_{j=0}^{2}{\sum }_{k=0}^{2}{\mu }_{ijk}\left[ Z{\text{arctan}}\left(\frac{X Y}{Z {R}_{ijk}}\right)- X{\text{ln}}\left({R}_{ijk}+Y\right)- Y\mathrm{ ln}({R}_{ijk}+X)\right]$$

where $X=\left(x-{x}_{i}\right), Y=\left(y-{y}_{j}\right), Z=\left(z-{z}_{k}\right),$ *i, j, k* are 1 and 2.

$${R}_{ijk}=\sqrt{{\left(x-{x}_{i}\right)}^{2}+{\left(y-{y}_{j}\right)}^{2}+{\left(z-{z}_{k}\right)}^{2}}$$ and $${\mu }_{ijk}={(-1)}^{i} {(-1)}^{j} {(-1)}^{k}$$

As for the components of GGT for this 2-3D prismatic body, one can directly take directional derivatives of the components above in the spatial domain. An alternative way which is mathematically less cumbersome is employing Fourier domain for calculation of the derivatives and then transforming the results back to the spatial domain.

### Depth constraint

Having an a priori depth to the COM (${z}_{0}$), we define a parameter,$f$, over the COM of a prismatic body, (x_0_, y_0_). The horizontal location of the COM (we call it target) could be attained from ${\Gamma }_{zz}$ in the case of LOP–PP category^13,15^, and from the logistic total horizontal gradient LTHG filter^18^ for the LOP-POP category. In fact, LTHG peaks over the edges of a POP. Therefore, based on the margins, the target could be marked. In this event, any point inside the horizontal trace of the POP, far from the edges, could be considered as the target.

6 $$f ={z}_{0} {\left[\frac{{\Gamma }_{zz}}{{{\text{g}}}_{z}}\right]}_{({{\text{x}}}_{0}, {{\text{y}}}_{0})}$$

Then two categories of models_ LOP–PP and LOP–POP _ are considered (Fig. 1a, b).

Depending on the assumed model, ${f}_{LOP-PP}$ and ${f}_{LOP-POP}$ are defined as:

7 $${\left(f\right)}_{LOP-PP}={\left({z}_{0}\right)}_{LOP-PP}{\left[\frac{{\Gamma }_{zz}}{{{\text{g}}}_{z}}\right]}_{({x}_{0}, {y}_{0})}$$

8 $${\left(f\right)}_{LOP-POP}={\left({z}_{0}\right)}_{LOP-POP}{\left[\frac{{\Gamma }_{{\text{zz}}}}{{{\text{g}}}_{{\text{z}}}}\right]}_{({x}_{0}, {y}_{0})}$$

$f$ at (x_0_, y_0_) is observed to vary from 1 to 2 for different models within LOP–PP category, and from 1 to 0.2 for various models within LOP–POP category.

At the same time, *I* for LOP–PP, and LOP–POP categories vary from 0 to 1 at the target. We examined the variations of $f$ obtained from (7) and (8) with respect to *I* for the two groups of models (Figs. 1c, d). When the body changes from LOP with *I* = *0* to PP with *I* = *1* (Fig. 1a), $f$ increases from 1 to 2 non-linearly (Fig. 1c). In contrasts, when the body changes from an LOP with *I* = *0* to a POP with *I* = *1* (Fig. 1b), $f$ decreases from 1 to 0.2 non-linearly (Fig. 1d). The variations of $f$ with respect to *I* is independent of the depth of the causative body and its real horizontal geometrical shape for each category. Therefore, if we gain the mathematical relation between $f$ and *I*, the depth of body from relations (7) and (8) could be estimated. Fitting 10^th^ order polynomials to $f$ in $f$-*I* curves in Figs. 1c, d, two categories of $f$ over the target at (x_0_, y_0_) are attained:

9 $${f}_{LOP-PP}=\sum_{i=0}^{10}{{ (P}_{i})}_{LOP-PP} {I({x}_{0}, {y}_{0})}^{10-i}$$

where P_LOP-PP_ = [2103.18992684381, − 9631.96402211124, 18,577.6251289147, − 19,588.2995049138, 12,248.4374659662, − 4593.09200836508, 983.430010323201, − 99.9187201174857, 0.120606818475533, 1.40856361966959, 1.01450450959620]

10 $${f}_{LOP-POP}=\sum_{i=0}^{10}{{ (P}_{i})}_{LOP-POP} {I({x}_{0}, {y}_{0})}^{10-i}$$

where P_LOP-POP_ = [6848.67493381295, − 36,658.5991267149, 84,416.3911603620, − 109,383.131515810, 87,587.6244287788, − 44,828.8689194104, 14,673.1097375456, − 2993.63766093542, 361.752513427095, − 24.3068510461594, 1.20590372220942].

The root mean square (RMS) error for ${f}_{LOP-PP}$ and ${f}_{LOP-POP}$ in Figs. 1c, d are, respectively, 0.004 and 0.010. Although the higher order of polynomials gives more precise estimations of $f$, the 10^th^ degree is precise enough to solve for the depth in this study.

Consequently, from relations (7) and (8), the depth of the anomalous mass could be estimated as:

11 $${\left({{z}^{\hat{\phantom{a}}}}_{0}\right)}_{LOP-PP} ={\left(f\right)}_{LOP-PP}\left[\frac{{{\text{g}}}_{z}}{{\Gamma }_{zz}}\right]$$

12 $${\left({{z}^{\hat{\phantom{a}}}}_{0}\right)}_{LOP-POP} ={\left(f\right)}_{LOP-POP}\left[\frac{{{\text{g}}}_{z}}{{\Gamma }_{zz}}\right]$$

Three endmembers of the models- LOP, PP and POP- are, respectively:

13 $$f=1\to {\left({{z}^{\hat{\phantom{a}}}}_{0}\right)}_{LOP} =\left(1\right) \left[\frac{{{\text{g}}}_{z}}{{\Gamma }_{zz}}\right]$$

14 $${f=2\to \left({{z}^{\hat{\phantom{a}}}}_{0}\right)}_{PP} =\left(2\right) \left[\frac{{{\text{g}}}_{z}}{{\Gamma }_{zz}}\right]$$

15 $${f=0.2\to \left({{z}^{\hat{\phantom{a}}}}_{0}\right)}_{POP} =\left(0.2\right) \left[\frac{{{\text{g}}}_{z}}{{\Gamma }_{zz}}\right]$$

From relations (13), (14) and (15), the coefficients 1, 2, and 0.2 are the same structural index, appeared in the final solutions of Euler deconvolution method^7^. Although structural index for POP is considered 0.5 in Euler method^7^, 0.2 seems to be a more precise measure in an extreme case of a 3D POP, where the horizontal dimensions of the body are large enough. Note that Euler deconvolution only considers the extreme cases, i.e., LOP, PP, and POP. Depending on *I*, our methodology, however, derives a spectrum of solutions where transitions between extreme shapes occur, meaning that the causative body is not limited to pure 2D or pure 3D but can also be 2–3D.

**Figure 2:**
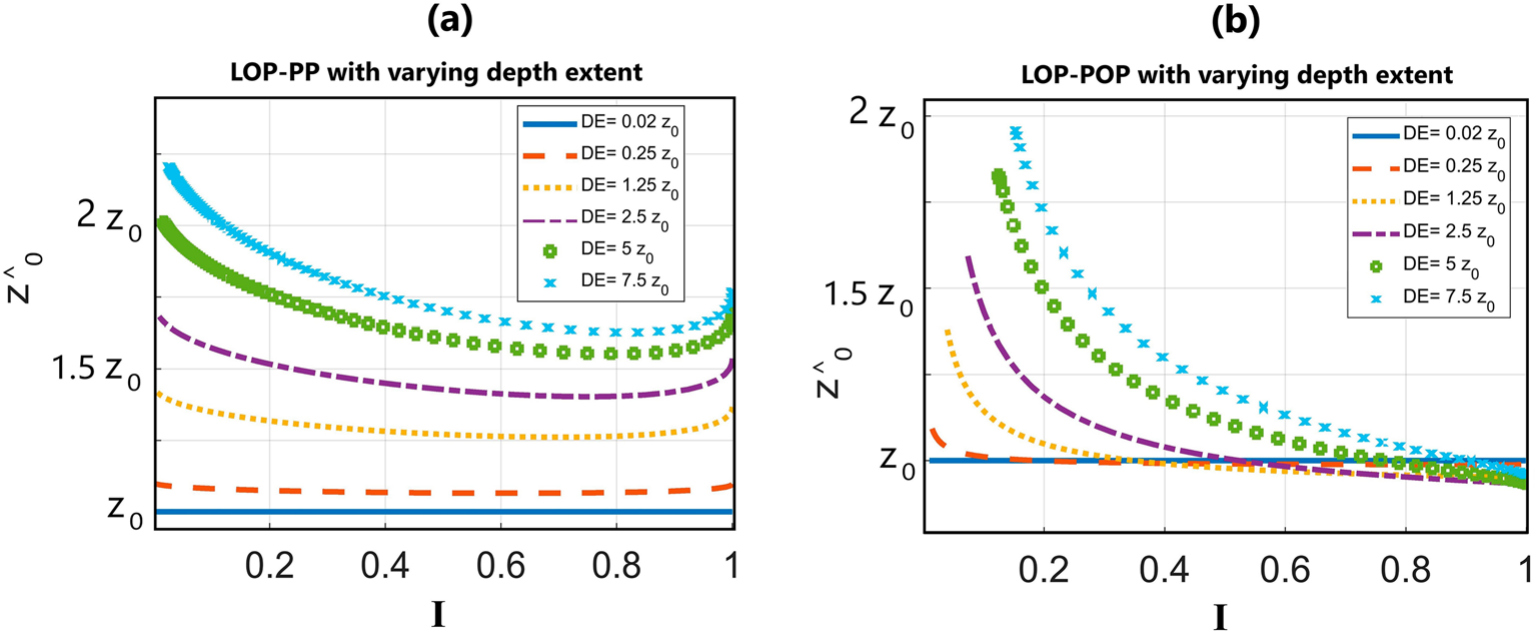
Variations of estimated depth (*z*^*^*^_*0*_) with respect to *I* and depth extent (DE) for (**a**) LOP-PP category, and (**b**) LOP-POP category.

We examined the behavior of *I* with respect to half-length $|\frac{\Delta y}{2}|=|\frac{L}{2}|$ along the strike when the observation point (OB) is on the y axis but shifts from the center to the sides of the LOP (Fig. 1e). At the center, OB = (0, 0), when $L\to 0$, *I* $\to$ 1. Increasing the length of the source $|\frac{L}{2}|$, *I* drops. Where $|\frac{L}{2}|$ goes to physical infinity (here $\mathrm{to }8{z}_{0}$), *I* approaches zero (Fig. 1f, blue curve). In the meantime, $f(I)$ falls from 2 to 1 as illustrated in Fig. 1c (blue curve). When the observation point is at $(x=0, y=2{z}_{0})$ (Fig. 1e), and $|\frac{L}{2}|$ grows from zero to $8{z}_{0}$, the body appears to be 2–3D for observation points $(x=0, y \mathrm{close to} 2{z}_{0})$ (Fig. 1f, red curve). Increasing $|\frac{L}{2}|$, *I* decreases to zero, implying the observer sees the body as a 2D structure. For farther observational points from the center, say, $(x=0, y=4{z}_{0})$, the transition from pure 3D to pure 2D occurs at $(x=0, y \mathrm{close to} 4{z}_{0})$ (Fig. 2b, yellow curve). The purple curve shows the observation point at $\left(x=0, y=6{z}_{0}\right).$ The dashed black line in Fig. 1f demonstrates a fixed value for the half-length of the LOP (5.5 ${z}_{0}$), but variable observation points. This means that if the half-length of LOP ($\frac{L}{2}$) equals 5.5 ${z}_{0}$, the observer at $\left(x=0, y=6{z}_{0}\right)$ calculates $I\approx 1$, while the other observers calculate $I\approx 0$. As a result, the observation point affects the calculation of *I*, thereby $f$ and *z*^*^*^_*0*_. It is of note that our methodology mandates the observation point to be within the horizontal extent of the body and far from the edges if the horizontal extent is wide.

### The effect of depth extent on the depth solutions

In the previous section, we derived a general formula for depth estimation of two different types of causative bodies, LOP–PP and LOP–POP, with small depth extents. In such cases, the estimated depth represented the depth to the center of mass.

We further investigated the impact of varying depth extent of the anomalous mass and the dimensionality indicator (*I*) on *z*^*^*^_*0*_ using relations (11) and (12), assuming that the top surface is buried at *z*_*0*_ (Fig. 2). When the LOP–PP category is extremely thin (depth extent = 0.02 *z*_*0*_), the estimated depth appears to be independent of variations in *I* (Fig. 2a). However, as depth extent increases, the *z*^*^*^_*0*_ becomes more reliant on *I*. Furthermore, Fig. 2a demonstrates that increasing depth extent also influences the average *z*^*^*^_*0*_. As depth extent approaches physical infinity (approximately 7.5 *z*_*0*_), the *z*^*^*^_*0*_ converges to ~ 1.75 *z*_*0*_.

For the LOP–POP category, we examined *z*^*^*^_*0*_ with varying depth extent and *I*, and the results are depicted in Fig. 2b. Similarly to the LOP–PP solutions, the *z*^*^*^_*0*_ for the LOP–POP category is dependent on *I*. However, the errors for LOP–POP solutions with larger *I* are smaller compared to the errors of LOP–PP solutions. As depth extent increases toward physical infinity, the average *z*^*^*^_*0*_ for LOP–POP solutions converges to ~ 1.25 *z*_*0*_.

### Simulated models in the absence and presence of noise

**Figure 3:**
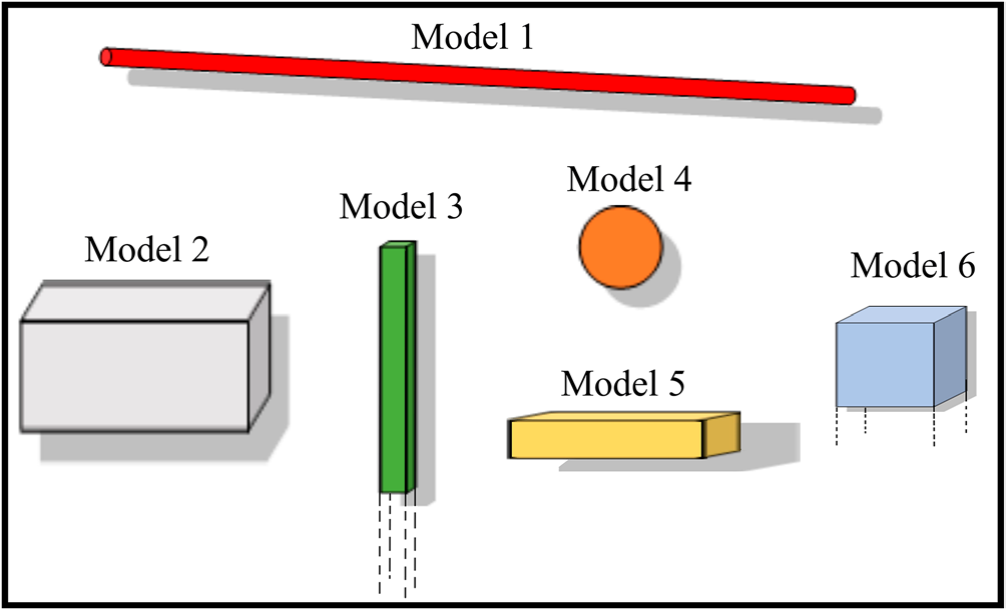
Model 1: Line of poles with Length of 40; Model 2: prismatic body with Width = 2, Length = 8, Depth extent = 4; Model 3 prismatic body with Width and Length = 1, Depth extent = 20; Model 4, point pole with radius 2, Model 5 prismatic body with Width = 1, Length = 6 and Depth extent = 2; Model 6 prismatic body with Width and Length = 40 and Depth extent = 60. The units for bodies’ dimensions are meter. The apparent size of the bodies is not representative of their real dimensions relative to each other. See Table 1 for information about the estimated model positions and *I*.

Here we consider simulated isolated and interfering pure 2D, pure 3D and 2-3D sources without and with 5% random Gaussian noise. The specification of the models and their positions are introduced in Fig. 3 and in Tables 1 and 2. At each case, we calculated the Normalized Root Mean Square Error (NRMSE) of the estimated location (Tables 1 and 2).

**Table 1:** Specifications of the isolated models along with their respective solutions.

Model	Dimensions (L,W,DE)	${r}_{0}$(m)	Noise = 0			Noise = 5%		
			${{r}_{0}}^{est}$(m)	*I*	NRMSE (%)	${{r}_{0}}^{est}$(m)	*I*	NRMSE (%)
Model 1	(40,0,0)	(0, 0, z_COM_ = 5)	(0, 0,5)	0.02	0.1	(0, 0.6, 4.57)	0.019	10.29
Model 2	(8,2,4)	(0, 0, z_COM_ = 6)	(0, 0, 5.54)	0.87	4.38	(0.3,0.4, 4.55)	0.77	14.74
Model 3	(1,1,20)	(0, 0, z_COTS_ = 2)	(0, 0, 3.72)	1	49.0	(0,0, 2.88)	0.83	25.42
Model 5	(6,1,2)	(0,0, z_COM_ = 3)	(0,0, 2.77)	0.65	4.33	(0.5,0, 2.53)	0.64	13.19
Model 6	(40,40,60)	(0,0, z_COTS_ = 3)	(0,0, 4.60)	1	30.80	(− 4.6, − 6.3, 4.87)	0.73	154.4

*L* is length, *W* is width and *DE* is depth extent, *COM* is center of mass, and *COTS* is center of top surface of a causative body. Grid unit is 0.1 m by 0.1 m, the model dimension and distances are in meter, and GM = 1 N m^2^ kg^−1^.

**Table 2:** Specifications of the interfering model 4 along with their respective solutions.

Model	GM ( N m^2^ kg^−1^)	${r}_{0}$(m)	Noise = 0			Noise = 5%		
			${{r}_{0}}^{est}$(m)	*I*	NRMSE (%)	${{r}_{0}}^{est}$(m)	*I*	NRMSE (%)
Three interfering Model 4, with equal densities and depths	1	(− 5,− 5, z_COM_ = 3)	(− 5, − 5, 2.96)	0.97	0.60	(− 4.9, − 5.2, 2.39)	0.80	12.35
		(0, 0, z_COM_ = 3)	(0,0, 2.95)	0.92	0.90	(0, 0.1, 2.35)	0.72	12.55
		(5, 5, z_COM_ = 3)	(5, 5, 2.96)	0.97	0.6	(5.1, 4.9, 2.41)	0.76	11.58
Three interfering Model 4 with equal densities and different depths	1	(− 5,− 5, z_COM_ = 3)	(− 5, − 5, 3.06)	0.99	1.17	(− 5, − 5, 2.53)	0.92	9.04
		(− 5, 5, z_COM_ = 2)	(− 5, 5,1.99)	0.99	0.04	(− 5.1, 5, 1.45)	0.73	16.09
		(5, 0, z_COM_ = 4)	(5,0,4.03)	0.98	0.5	(4.8,0.3, 2.75)	0.78	18.69
Three interfering Model 4 with different densities and depths	1	(− 5 − 5,z_COM_ = 3)	(− 5, − 5, 3.14)	0.99	2.74	(− 5.1,− 5.3, 2.22)	0.76	16.06
	1.5	(− 5, 5,z_COM_ = 2)	(− 5, 5, 1.99)	0.99	0.07	(− 5.2, 4.9, 1.49)	0.82	16.02
	2	(5, 0, z_COM_ = 4)	(5, 0, 4.01)	0.99	0.23	(4.8, 0.2, 2.91)	0.82	16.2

Grid unit = 0.1 m by 0.1 m, the model dimension and distances are in meter, and the radius of individual model 4’s is 2 m.

The NRMSE is defined as follows^33^:

16 $$NRMSE=\frac{1}{{z}_{0}}\sqrt{\frac{{({{x}^{\hat{\phantom{a}}}}_{0}-{x}_{0})}^{2}+{({{y}^{\hat{\phantom{a}}}}_{0}-{y}_{0})}^{2}+{({{z}^{\hat{\phantom{a}}}}_{0}-{z}_{0})}^{2}}{N}}\times 100$$

where ${{r}^{\hat{\phantom{a}}}}_{0}=({{x}^{\hat{\phantom{a}}}}_{0},{{y}^{\hat{\phantom{a}}}}_{0},{{z}^{\hat{\phantom{a}}}}_{0})$ and ${r}_{0}=({x}_{0}, {y}_{0}, {z}_{0})$ are the estimated COM/COTS (depending on the depth extent) and real position of the COM/COTS, respectively. *N* is the number of elements considered in relation (16). Exploring on a grid map requires *N* = *3* (for x, y, and z components), and on a profile, *N* = *2*.

Table 1 presents an overview of various isolated models and their associated parameters, specifically focusing on the estimation of a COM and COTS, denoted as “*r*^*^*^_*0*_”. It investigates how the accuracy of these estimations varies under different noise conditions. The dimensions and distances in Table 1 are in meter.

### Model 1:

Line of Poles (LOP) with length of 40 m, and COM coordinates at (0, 0, z = 5 m). Under noise-free condition, Model 1 exhibits a remarkably accurate estimation of “* r*^*^*^_*0*_” with a low NRMSE of 0.1. However, when noise is introduced, the NRMSE increases to 10.29%, indicating a reduction in estimation accuracy. *I* in both conditions indicates an almost pure 2D body.

### Model 2:

Prismatic body with Length = 8 m, Width = 2 m, Depth extent = 4 m, and COM coordinates at (0, 0, z = 6 m). *I* with and without noise are, respectively, 0.77 and 0.87, implying that the model is a 2-3D body. Although the effect of large depth extent in model 2 may reduce the accuracy of the solutions, they are still reasonable estimates of COM.

### Model 3:

Prismatic body with Length = 1 m, Width = 1 m, Depth extent = 20 m, and COTS coordinates at (0, 0, z = 2 m). Despite a significant depth extent, the solutions estimate the COTS in this model well ((0,0, 3.72 m) in noise-free condition versus (0,0, 2.88 m) in noisy condition).

### Model 5:

Prismatic body with Length = 6 m, Width = 1 m, Depth extent = 2 m, and COM coordinates at (0, 0, z = 3 m). The depth estimations in noisy and noise-free states are quite close (2.53 m against 2.77 m, respectively). Similarly, *I* values are calculated 0.64 and 0.65, in the order.

### Model 6:

A prismatic body with dimensions of Length = 40 m, Width = 40 m, Depth extent = 60 m, representing a Plane of Poles with physically infinite depth extent (60 m). The coordinates of the COTS are (0, 0, z = 3 m). *I* decreases from 1 in noise-free state to 0.73 in noisy state. Though in presence of noise the NRMSE increases notably from 30.80% to 154.4%, one should notice that a major contribution to this error comes from the horizontal components of the estimated COTS, (− 4.6 m, − 6.3 m, z = 4.87 m).

Table 2 provides an overview of different scenarios involving three interfering “Model 4” configurations (see Fig. 3). It evaluates the estimation of the parameters *I* and ${{r}^{\hat{\phantom{a}}}}_{0}$ under two noise conditions, specifically no noise and 5% noise.

#### Three interfering model 4 with equal densities and depths

For this set of scenarios with equal densities (GM = 1 N m^2^ kg^−1^) and equal depths (z_COM_ = 3 m), the ${{r}^{\hat{\phantom{a}}}}_{0}$ values under Noise = 0 are relatively close and exhibit low NRMSE values. In presence of noise, however, the solutions become more inaccurate with NRMSE of about 12% for individual bodies.

#### *Three interfering model 4 with equal densities but different depths*:

In this case, GM = 1 N m^2^ kg^−1^ for all three interfering bodies but the z_COM_ are different (3 m, 2 m, 4 m). The ${{r}^{\hat{\phantom{a}}}}_{0}$ values under Noise = 5% lose their great accuracy in comparison with the noise-free condition. Consequently, NRMSE values increase in noisy state, but the estimated depths still show a reasonable level of accuracy.

#### *Three interfering model 4 with different densities and depths*:

These scenarios involve both different densities GM = 1, 1.5, and 2 N m^2^ kg^−1^ and different depths, z_COM_ = 3 m, 2 m, 4 m. Although in this state the depths and densities are variable, the solutions yield acceptable results, with NRMSE values comparable with the previous states.

**Figure 4:**
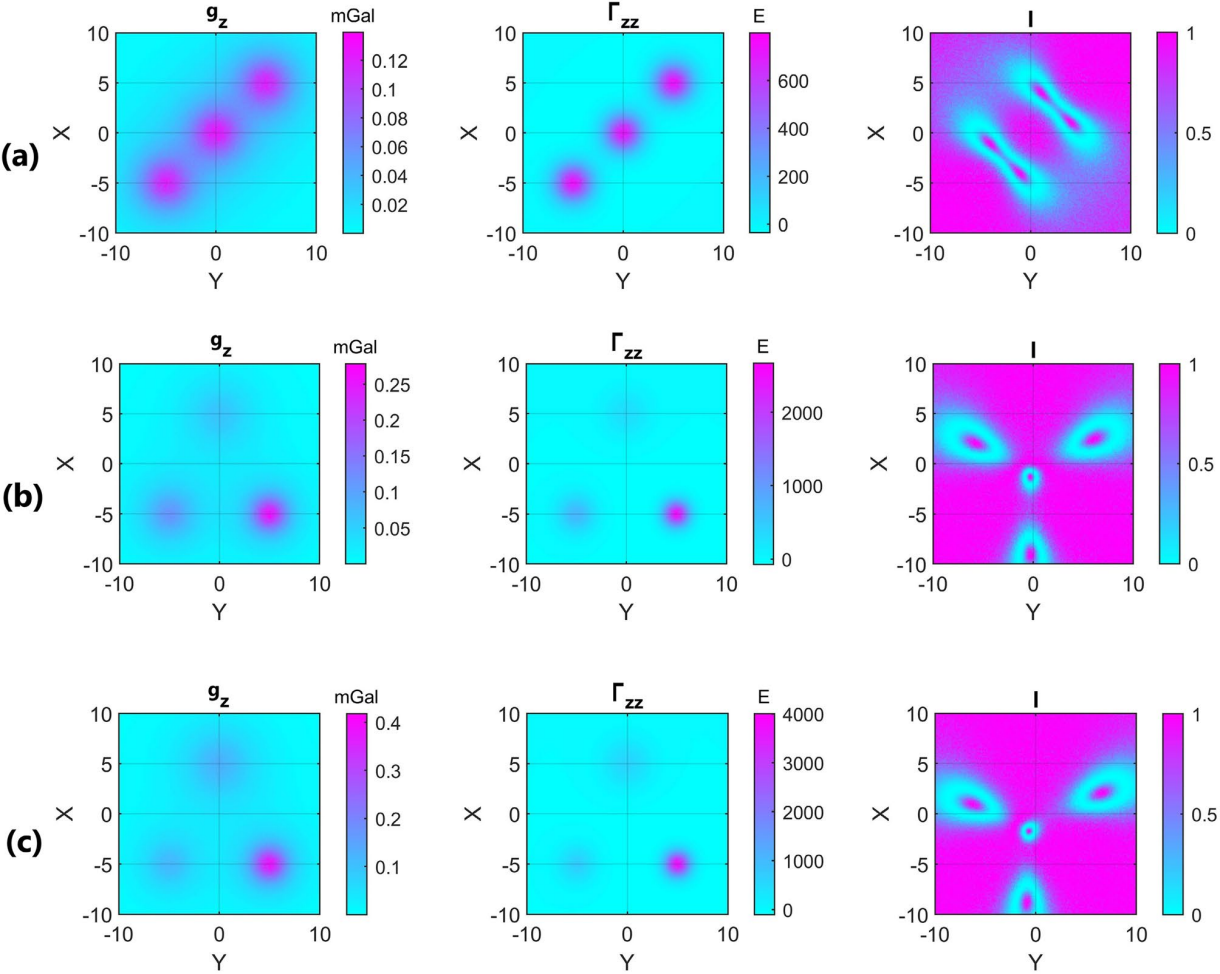
From left to right: ${g}_{z}, {\Gamma }_{zz}, I$ in presence of 5% random Gaussian noise for (**a**) three interfering model 4 with equal densities (GM = 1 N m^2^ kg^-1^) and depths (z_COM_ = 3 m); (b): three interfering model 4 with equal densities (GM = 1 N m^2^ kg^−1^), but different depths (z_COM_ = 3, 2, 4 m); and (c) three interfering model 4 with different densities (GM = 1, 1.5, 2 N m^2^ kg^−1^) and different depths (z_COM_ = 3, 2, 4 m). For more information about the models see Table 2.

Figure 4 indicates *g*_*z*,_ *Γ*_*zz*,_ and *I* for the three interfering configurations mentioned above in presence of 5% random Gaussian noise. *Γ*_*zz*_*,* pinpoints the target, and *I* at the target points delineates the dimensionality of the interfering spherical sources. One can see that the interference of the bodies distorts the *I* signature significantly in the vicinity of the targets. Nevertheless, *I* values are safe from this distortion exactly at the targets. It is deduced from Fig. 4 that determination of the target points is of paramount importance, and *I* values farther from the target points can make significant errors in our methodology.

## Workflow

1. The gravity anomaly ($${g}_{z})$$ and GGT components and invariants are calculated. Note that the units of $${g}_{z}$$ and $${\Gamma }_{zz}$$ are usually milligal and Eotvos, respectively. Therefore, they should be converted to their SI units for the subsequent analysis. 1milligal = 10^–5^ m/s^2^ in SI, and 1 Eotvos = 10^–9^ s^-2^ in SI.
2. The regional field should be eliminated from ${g}_{z}$. Usually, this could be done by subtraction of a fitting polynomial to ${g}_{z}$ map, in xy plane. The order of the polynomial is set by the user.
3. For the target delineation in LOP-PP category, maximum of ${\Gamma }_{zz}$ is marked.
4. For the target delineation in LOP-POP category, the points interiors of the causative body, far from the edges are used. The edges could be delineated from LTHG filter.
5. The dimensionality indicator $(I=-\frac{{{(I}_{2}/2)}^{2}}{{{(I}_{1}/2)}^{3}})$ is calculated for the target.
6. Based on the horizontal extent, derived from gravity parameters, in steps “3” and “4”, either ${f}_{LOP-PP}$ or ${f}_{LOP-POP}$ is considered at the target from Eq. (9) or (10).
7. From relations (11) or (12), the depth is calculated.

## Application to real data

The efficiency of our proposed method was probed and compared with the Euler deconvolution method on the lunar surface. The calculation of the Bouguer anomaly involves considering a rock density of 2560 kg/m^3 pertaining to the upper crust^24,36^. To obtain the residual gravity field, we conducted a subtraction of a 3rd order polynomial, that was fitted to the Bouguer anomaly, from the Bouguer anomaly data itself. The choice of the fitted polynomial is subjective; usually, when the exploration area is large enough to comprise numerous positive and negative anomalous structures with an undulating and non-planar regional field trend, opting for higher orders, i.e., 2 or 3, of the polynomial yields a more realistic estimation of the individual anomalies.

Due to the abundance of impact craters on the Moon, its earliest history (~ the first 700 million years) is barely preserved^40^. However, the Bouguer anomaly and its derivatives with the employed resolution can reveal numerous subsurface structures. Andrew-Hanna et al.^24^ identified a number of huge linear gravity anomalies as pre-Nectarian to Nectarian intrusive dike-like structures, which originate from both magmatism and lithospheric extension on the planet. In addition to the large-scale linear constructs, ${\varvec{\Gamma}}$ components demonstrate some other structures that are related to impact basins, as well as composition and porosity variations over the lunar crust.

**Figure 5:**
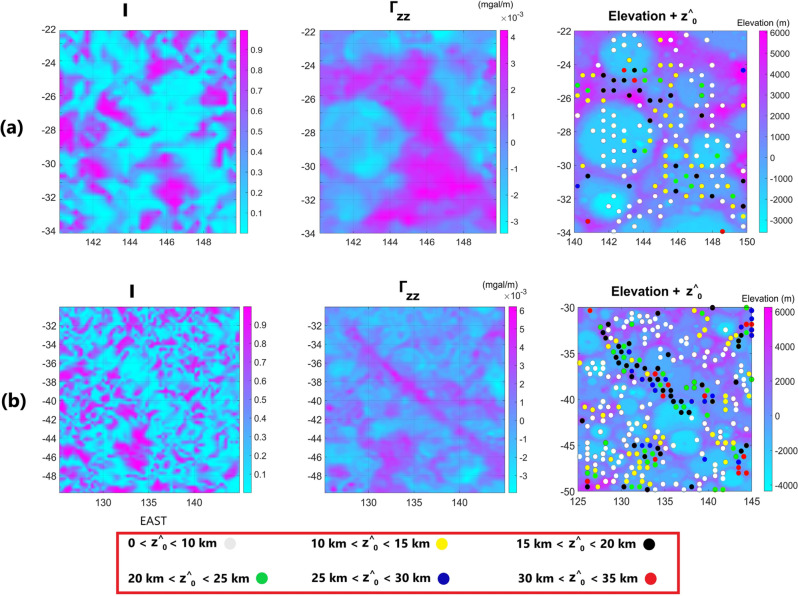
(**a**, **b**) from left to right; *I*, ${\Gamma }_{zz}$*,* and *z*^*^*^_*0*_ fitted on elevation, for two regions on Moon.

Figure 5 illustrates, from left to right, *I*, ${\Gamma }_{zz}$, and *z*^*^*^_*0*_ overlain on the elevation map, for two regions on the Moon. *I* and ${\Gamma }_{zz}$ in Figs. 5a and 5b show enormous linear structures that are attributed with mafic igneous intrusions rising from the upper mantle with higher average density than their vicinities^24^. *I* in location of these structures is close to zero (indicating 2-dimensionality). ${\Gamma }_{zz}$ depicts large linear values although this parameter in Fig. 5a is much broader than in Fig. 5b. The estimated solutions for these features could be credited to their top depth because of their likely large depth extent. We classified the depth solutions into 6 intervals from zero (over the reference ellipsoid) to 35 km. The average *z*^*^*^_*0*_ is higher for the solutions occurring over the dike-like structures (> 10 km). This is more pronounced for Fig. 5b, where the average depth to the top surface was estimated around 26 km by Andrew-Hanna et al.^24^. The solutions over the impact basins and other areas, having composition and/or porosity variation signatures, are shown to be shallower (< 10 km). In case of large impact basins, this could be because of mantle upwarps and/or brecciation which occur nearer to the surface. However, in the case of small impact basins and areas with composition/porosity variations, the impact gardening, brecciation and superficial faulting and fracturing are more likely agents. As a result, it is reasonable for the solutions in these areas to be shallower. It is necessary to see if these solutions could be validated by other methodologies; Thus, we implemented the well-known Euler deconvolution method in the explored areas shown in Fig. 5 to see if our solutions are consistent with them or not.

### Comparison with euler deconvolution

**Figure 6:**
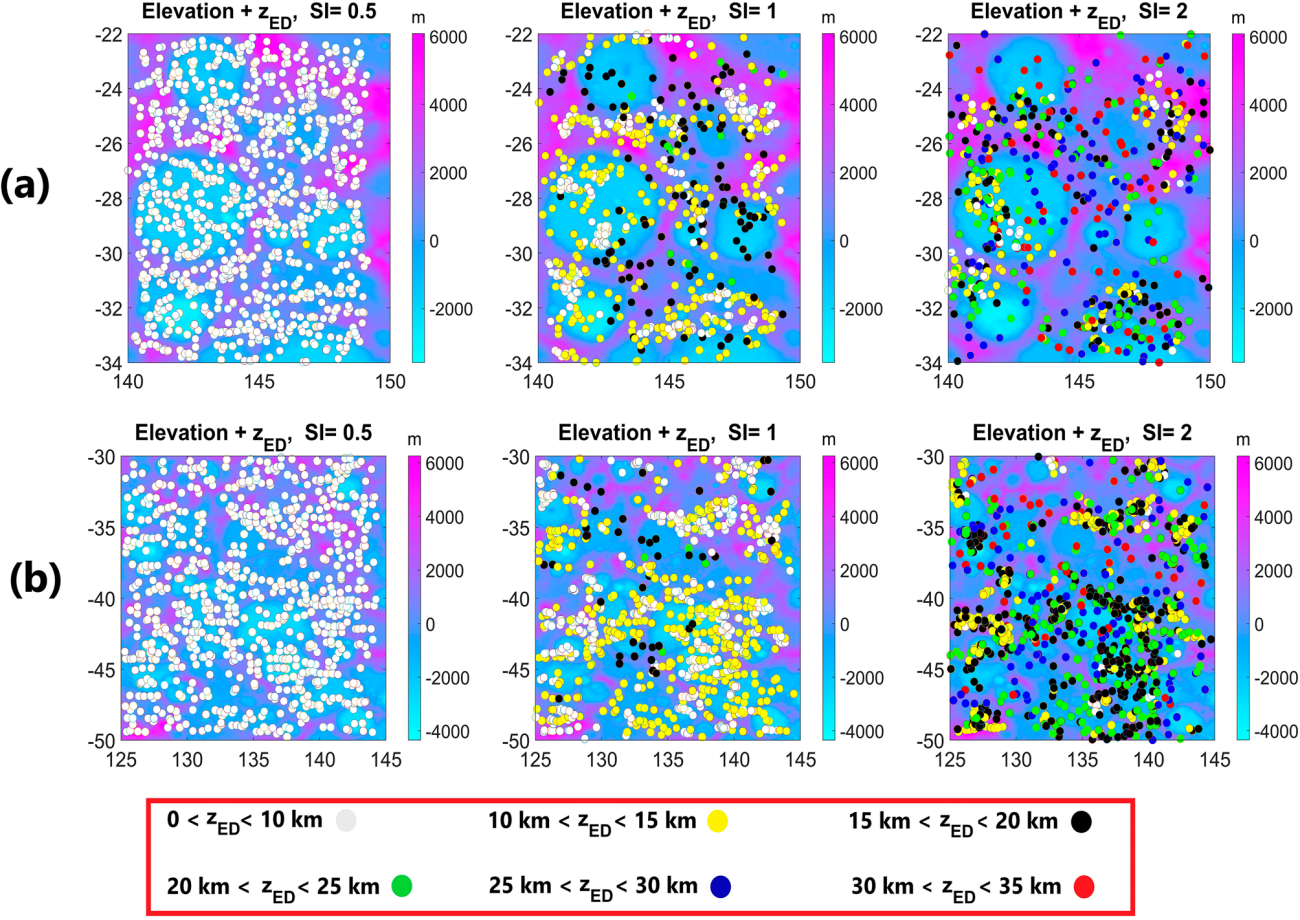
(**a**, **b**) depths attained from Euler Deconvolution method (z_ED_) fitted on elevation for structural indices (SI) from left to right; 0.5, 1, and 2, for two regions on Moon.

Figure 6 (a, b) illustrate the depth estimations (z_ED_) obtained using the Euler deconvolution method^7^ with three different structural indices. From left to right, the structural indices are represented as SI = 0.5, 1, and 2, corresponding to POP, LOP, and PP, respectively.

The Euler deconvolution technique is designed to examine three limiting cases (POP, LOP, and PP). Therefore, it is not feasible to employ varying structural indices within a single map. SI = 0.5 sets the minimum depth range, and SI = 2 determines the maximum depth range. The final solution for each area is chosen based on a priori geological information and the interpreter’s judgment.

In this process, a sliding window of 30 km by 30 km, comprising 9 data points, was used. To enhance the reliability of the calculations, solutions with ${z}_{ED}<10 (SI)({\sigma }_{z})$ were discarded, as recommended by Thompson^7^. Here, ${\sigma }_{z}$ represents the standard deviation of the estimated depth (z_ED_), calculated from the covariance matrix of the estimated model parameters^41^.

A comparison between Figs. 5 and 6 confirms that our solutions fall within the depth ranges calculated by Euler deconvolution, which typically range from 0 to 35 km (see Figs. 5 and 6). It is worth noting that Euler deconvolution provides only a rough estimate of the depth solutions due to the fixed nature of SI in its algorithm. In contrast, our method has the capability to distinguish between different SI values at each data point. Therefore, we believe that our method offers higher accuracy and reliability in depth estimation.

## Conclusion

We designed a novel method for detecting the depth of a causative body based on its relative horizontal dimensions, as indicated by the dimensionality indicator (*I*). The method categorizes causative bodies based on their horizontal spread, distinguishing between those falling within the line of poles and point pole (LOP–PP) category and those within the line of poles and plane of poles (LOP–POP) category, and introduces two types of solutions accordingly.

The introduced method estimates the depth of anomalous bodies with any dimensionality and, in limiting cases, becomes similar with the well-known Euler Deconvolution method. When the depth extent of a body is limited and small, the estimated depth (*z*^*^*^_*0*_) corresponds to the center of mass, while for bodies with large depth extent, the *z*^*^*^_*0*_ relates to the center of top surface.

The *z*^*^*^_*0*_ is influenced by both the depth extent and the dimensionality of the causative body. As the depth extent increases, the impact of *I* on the estimated depth becomes more pronounced. Additionally, the behavior of *z*^*^*^_*0*_ varies between the LOP–PP and LOP–POP categories, with LOP–POP solutions exhibiting lower errors for larger values of *I*.

The method is applied to defined synthetic isolated and interfering sources, both with and without noise. Once the stability of these solutions was achieved, we applied this method to lunar data that showed the presence of two significant linear structures. The results are aligned with the inferred geological information, validating the effectiveness of the approach.

Compared with some other techniques like Euler deconvolution method, our methodology presents more accurate estimates, and our solutions occur within those of Euler deconvolution which sets the upper and lower boundaries of the depth solutions. It requires fewer assumptions about the geology, i.e., limits assumptions to whether the gravity signal arises from a body within the LOP–PP category or the LOP–POP category. Additionally, to estimate the depth, only one data point is necessary. This new method can estimate the depth of anomalous causative bodies across a wide range of dimensionality from 2 to 3D.

Furthermore, our method, while straightforward, and not mathematically complicated provides a robust and efficient means for depth estimation of anomalous constructs, offering valuable insights into subsurface geology.

## Data Availability

The datasets generated and/or analysed during the present study are available in the [Planetary Data System (PDS)] repository, [https://pds-geosciences.wustl.edu/]. Matlab-based Graflab software was used for calculation of the Marussi tensor components.
